# Insights from a model based study on optimizing non invasive brain electrical stimulation for Parkinson’s disease

**DOI:** 10.1038/s41598-024-52355-2

**Published:** 2024-01-30

**Authors:** Maryam Sousani, Saeid R. Seydnejad, Maryam Ghahramani

**Affiliations:** 1 Faculty of Science and Technology, University of Canberra, Bruce, Canberra, 2617 ACT Australia; 2 Department of Electrical Engineering, Shahid Bahonar University of Kerman, Pajoohesh Sq., Kerman, Kerman Iran

**Keywords:** Parkinson's disease, Biomedical engineering, Dynamical systems

## Abstract

Parkinson’s Disease (PD) is a disorder in the central nervous system which includes symptoms such as tremor, rigidity, and Bradykinesia. Deep brain stimulation (DBS) is the most effective method to treat PD motor symptoms especially when the patient is not responsive to other treatments. However, its invasiveness and high risk, involving electrode implantation in the Basal Ganglia (BG), prompt recent research to emphasize non-invasive Transcranial Electrical Stimulation (TES). TES proves to be effective in treating some PD symptoms with inherent safety and no associated risks. This study explores the potential of using TES, to modify the firing pattern of cells in BG that are responsible for motor symptoms in PD. The research employs a mathematical model of the BG to examine the impact of applying TES to the brain. This is conducted using a realistic head model incorporating the Finite Element Method (FEM). According to our findings, the firing pattern associated with Parkinson’s disease shifted towards a healthier firing pattern through the use of tACS. Employing an adaptive algorithm that continually monitored the behavior of BG cells (specifically, Globus Pallidus Pars externa (GPe)), we determined the optimal electrode number and placement to concentrate the current within the intended region. This resulted in a peak induced electric field of 1.9 v/m at the BG area. Our mathematical modeling together with precise finite element simulation of the brain and BG suggests that proposed method effectively mitigates Parkinsonian behavior in the BG cells. Furthermore, this approach ensures an improvement in the condition while adhering to all safety constraints associated with the current injection into the brain.

## Introduction

PARKINSONS’ Disease (PD) is the second most common neurodegenerative disorder of the central nervous system after Alzheimer’s disease^1^. The main factor in the onset of the PD is degeneration of the dopaminergic neurons in Basal Ganglia (BG) structure located in the mid-brain^2^. The task of BG in the brain is controlling and also learning the movement. BG receives its input from the cortex and sends its output to the thalamus. It is believed that the PD induces pathological oscillations in different blocks of the BG-thalamus neuronal network and also reduces the functionality of the thalamocortical (TC) relaying of sensorimotor signals^3^. These impairments in the brain caused by PD, lead to different physical motor and non-motor cognitive symptoms in patients.

The main motor symptoms of PD are tremor, rigidity, bradykinesia, and postural instability^4^. Deep brain stimulation (DBS) is a neurosurgical treatment for severe physical symptoms of PD^5^. While DBS is an effective method in treating PD symptoms, it has some drawbacks. Deep brain stimulation is an invasive method that includes the risk of surgical complications and hardware dysfunction such as lead breaking or battery failure. Also, its implantation procedure is very expensive^6^. Therefore, finding alternative safer methods for the treatment of PD motor symptoms is of great importance.

Recent research has highlighted the potential benefits of non-invasive brain stimulation, such as transcranial electrical stimulation (TES) and transcranial magnetic stimulation (TMS). For instance, as reported in a systematic review by George^7^, daily prefrontal TMS treats acute depression in treatment-resistant unipolar patients. Additionally, Epstein et al.^8^, applied 10 Hz repetitive TMS to 14 patients diagnosed with PD and comorbid treatment-resistant depression. Highly significant improvements in depression scores were reported a few days after treatment. Two extensively employed TES methods include alternating (TACS) and direct current (TDCS). The mechanisms of action of TACS and TDCS are substantially different^9^. tDCS, for instance, is believed to modify neuronal firing thresholds by adjusting the resting membrane potentials of neurons, either enhancing or reducing them^10^. However, the application of sinusoidal current in tACS implies that the overall membrane potential remains unchanged. Consequently, the mechanism behind TACS is likely to be rooted in synchronizing existing cortical activity rather than inducing specific changes in cortical excitability at a local level^10,11^. The tACS and tDCS methods are used for controlling and management of different neurological disorders such as dementia, dystonia, and epilepsy^12,13^. In these neuro-modulation methods, the stimulating current is applied mainly via two large surface electrodes on the scalp. The ability to modulate the cortical excitability and the positive results obtained in motor recovery in chronic stroke patients prompted the researchers to examine tDCS in PD treatment. Most studies that have evaluated the Unified Parkinson’s Disease Rating Scale (UPDRS) in PD patients before and after tDCS, reported an improvement in motor function properties^14–16^. Combination of tDCS and conventional gait rehabilitation therapy has been recognized to produce more favorable outcomes^17^.

In all studies using TES for PD patients so far, two large electrodes are placed on the scalp and the injected current is limited due to safety constraints^18^. The shunting effect of the scalp decreases the amount of current intensity delivered to the targets in deep layers of the brain and there is no control to guide the current to any specific area just by two electrodes. By changing the position of electrodes on the scalp, current flow and therefore its effect on the brain can change. However, the injected current has very limited capability to reach the desired target in this method^19,20^. Therefore, there has been increasing interest in research for finding new methods of non-invasive electrical stimulation and enhancing their effectiveness.

Recently high-definition TES (HD-TES) which uses an array of small electrodes has been proposed for an effective current focalization^21^. This method increases the degrees of freedom in controlling the flow of current. It can be employed in a mathematical framework such as a beamforming scheme to conduct the current to a specific brain target area while preserving the safety constraints^22^. Park et al.^23^ investigated the effect of multiple small electrodes and were able to shape the current to the target area. In a study by Dmochowski et al.^24^, using HD-TES, a beamforming approach was proposed to achieve an optimal electrode current distribution and to focalize the electric field in a given target area while preserving current constraints. In order to find optimal electrode stimulus patterns for HD-TES, Guler et al.^25^ used a convex optimization method to maximize the current density in a particular direction. Dmochowski et al.^26^, showed that using the focalized stimulation with HD-TES, stroke rehabilitation patients scored 38% higher performance on their behavioral task. In a study by Wang et al.^27^, using several electrodes, a non-invasive DBS stimulator was developed with precise positioning and real-time monitoring of bio-impedance. Based on the findings of a pilot study by Na et al.^28^, it appears that utilizing multichannel tDCS on the motor cortex of the leg during treadmill gait training is a safe and effective approach for enhancing gait velocity in PD patients. Although all these HD-TES approaches with different focalization methods have led to better quality stimulation, they have not been used in an adaptive stimulation approach.

Recently, there has been a vital need for non-invasive adaptive stimulation^29^. In other words, as quoted by some clinicians, they need some ways to be able to “listen into the brain” and have the brain “tell us what it needs”^29^. A highly promising but under-explored strategy is to “close the loop” by adjusting the stimulation as a function of measured brain activity which is known as feedback control. Closed-loop stimulation has been widely used for DBS method to find appropriate stimulation parameters in order to decrease side-effects of the stimulation^30^. Despite the importance of non-invasive closed-loop stimulation and its proven efficacy with more personalized stimulation parameters, there are only few studies in the literature focusing on this method. For instance, Del Felice et al.^31^, used Electroencephalography (EEG) power spectra maps to find individualized electrode positions and frequency for tACS. The authors presented data to support the effectiveness of personalized tACS in combination with physical therapy for improving both motor and cognitive symptoms in individuals with PD. Brittain et al.^32^ used a rhythmic transcranial current stimulation method for PD tremor suppression. They considered the measured tremor of the hand as a feedback controller. Using this feedback, authors regulated the stimulation parameters. Results of this report show that using phase cancellation tACS, they achieved a reduction in resting tremor amplitude. While the efficacy of closed-loop tACS has been proved in this report, additional studies are required to understand the key tenets of this method more comprehensively.

This simulation-based study aimed at proposing a novel non-invasive closed-loop TES method for enhancing the Parkinsonian behavior of the BG cells’ firing pattern. Using a current focalization method, the delivered current to deep layers of the brain especially the BG area is maximized which leads to a change in the oscillatory behavior of BG cell activation patterns. In the proposed method, firstly, a group of small electrodes is placed on the scalp in the simulation area. Then using an adaptive closed-loop method for our constraint minimization algorithm, the amount of each electrode current is adjusted in each iteration. Finally, after a series of iterations, the injected current is concentrated on the BG area to affect neural activation patterns. The first contribution of this study is the development of a closed-loop stimulation method using non-invasive TES to enhance the Parkinsonian behavior in firing pattern of the BG cells. The second contribution is the use of adaptive beamforming techniques for optimization of electrode number and location. This could help to improve the accuracy in different environments and with different parameters. The third contribution is the development of analytical and perturbation-based methods for calculating gradients to measure changes in firing patterns based on the applied stimulation. This could help to optimize the stimulation parameters and improve its effectiveness in real-life environments. The fourth contribution is the use of two different head models to validate the algorithm in different environments and with different parameters. This could help to ensure that the method is effective across a range of individuals and scenarios in real-life experiments. Finally, the fifth contribution is a comparison of the results obtained from this study with other studies in the field that have similar parameters of the head model and stimulation. This could help to establish the effectiveness of the proposed method and its potential advantages over other methods.

## Methodology

The objective of this study is to employ a novel method aimed at modulating the aberrant firing patterns of cells within the BG area. This is achieved through the application of non-invasive stimulation specifically targeting the STN cells, addressing the abnormal firing patterns observed in Parkinson’s disease. Although tACS is the stimulation technique used in this study, the proposed method is not limited to tACS and can be used with tDCS as well. The primary reason for using tACS is to substitute the stimulation parameters with DBS in the mathematical model of BG. However, in real-life situations where different feedback such as hand tremor or EEG is available, direct current can be used for stimulation. For the aim of this study, three fundamental steps are required: (a) applying a current focalization algorithm to conduct the current injected into the scalp by an array of electrodes to the BG, (b) monitoring the effect of the current stimulation on the activation patterns of BG cells, (c) implementing a closed loop mechanism to orchestrate the current adjustment and changes of the activation pattern in an adaptive manner. The current focalization section focuses on three fundamental steps. Firstly, we describe the exploration of head models and the utilization of the beamforming method to achieve current focalization in the proposed technique. Secondly, we provide an explanation of the behavior of cells in the BG mathematical model and how feedback is incorporated into the study. Thirdly, the closed-loop method is expanded through the use of a constraint minimization algorithm to identify the most effective current injection pattern for affecting the activation patterns of BG cells. This section also introduces both an analytical and a perturbation-based method for calculating the gradient vector.

### Current focalization

To focalize the current onto the BG area, two steps are needed: (1) head modeling and (2) beamforming. Using head modeling the current distribution in the conductive layers of the brain can be shown. Then, an appropriate beamforming algorithm for current focalization needs to be implemented. The two steps are explained in detail in the following.

#### Head modeling

Distribution of the current and its induced electric potentials through the brain depends on the conductivity and the structure of different brain tissues. In this study two head models are used to develop our proposed method; a realistic Magnetic Resonance Imagery (MRI) based heterogeneous head model and a simple homogeneous spherical model^33^.

The MRI-based heterogeneous head model provides a realistic environment for a thorough characterization of the proposed method and evaluation of its performance in a realistic scenario. For this model, the MRI information of a patient is divided into five different parts using the SPM software package (2017, version8). The five parts in this model include scalp, skull, cerebrospinal fluid (CSF), gray matter, and white matter. Next, a 3D model including the geometry of the head and electrodes are built using the Simpleware ScanIP software (Synopsys, Inc. 2017). ScanIP is a 3D image processing and model generation software to visualize, analyze, quantify, segment, and export 3D data from MRI. Electrodes are modeled as metal cylinders and added to the head model using the Simpleware CAD Module (Synopsys, Inc. 2017). Since the current is injected through external electrodes, the divergence of the current density $\left( {\nabla .{{\textit{J}}}} \right)$ inside the brain is zero. Therefore, the potential distribution in the brain can be obtained by Laplace’s equation,

1 $$\begin{aligned} \nabla .{\textbf{J}} = \nabla .\left( {\sigma {\textbf{E}}} \right) = - \nabla .\left( {\sigma \nabla {\textbf{V}}} \right) = 0 \end{aligned}$$

where $\sigma$ ,***V*** denote conductivity and electric potential while ***J***, ***E*** represent the vectors of the current density and electric field, respectively. To solve (1) a numerical method of the finite element method (FEM) is used. This approach divides a conductive volume into a large number of small cells called elements. Each element is connected to its neighboring elements through multiple nodes. This process is called meshing and is handled using Simpleware FE Module (Synopsys, Inc. 2017). In this study we chose 17,843,965 tetrahedral elements for the realistic head model which is comparable to other studies in the field^23,24^. To obtain the current density, the COMSOL MultiPhysics software (Version 5.0, 2017) was employed to process the FEM of the head model.

The simple homogeneous spherical model is computationally simple and gives some insight into the brain current distribution. This simple spherical head model consists of four layers: scalp, skull, CSF, and the brain tissue. These layers are in the form of concentric spherical shells with different conductivities. The gray and white matters are considered as one layer for simplicity and they are modeled by their average conductivities which is called brain tissue. In a similar fashion to Datta et al.^34^, the simple head model in this study includes 130 306 tetrahedral FEM elements.

#### Beamforming

Creating a focalized current in a given target by injecting electrode currents can be treated similar to beamforming techniques utilized in the context of sensor array signal processing^24^. Beamforming is a type of radio frequency (RF) management in which a wireless signal is directed toward a specific receiving device. Beamforming works differently depending on its type or implementation. Multiple antennas in close proximity send out multiple signals at different times. A beamforming tower or router determines the best path for the signals to take to reach the client device. Beamforming shapes the RF beam as it traverses a physical space. Although the beamforming technique is a widely used method in telecommunications and signal processing, there are only few studies using beamforming for electric current focalization^24,26,35^.

Our proposed method uses a beamforming current focalization method in line with the method proposed by Dmochowski et al.^24^. The approach they employ relies on minimizing the error between the desired electric fields and the electric fields induced at the target location, using the method of Least Squares (LS). In this simulation based study, we use the behavior of the BG cells firing pattern as feedback for our adaptive algorithm in order to enhance this pattern. In this adaptive beamforming technique, electrode currents are adjusted continuously to focalize the current in a special part of the brain responsible for generation of abnormal firing pattern. The injected electrode currents are adjusted by the algorithm such that they create a focal current point in the BG to affect this firing pattern.

### BG mathematical model

Development of the PD and generation of its motor symptoms are associated with changes in neuronal firing patterns within the BG cells^36^. Abnormally irregular firing patterns of motor units in PD consist of pairs of spikes. While in non-Parkinsonian participants a regular spike pattern is observed at different nodes within the BG, the pattern changes to a bursting one in PD patients^37,38^. In neuro-feedback closed-loop studies, these changes of neuronal firing pattern has been considered as a function of measured brain activity to distinguish between PD and non-PD states^29,36^.

**Figure 1:**
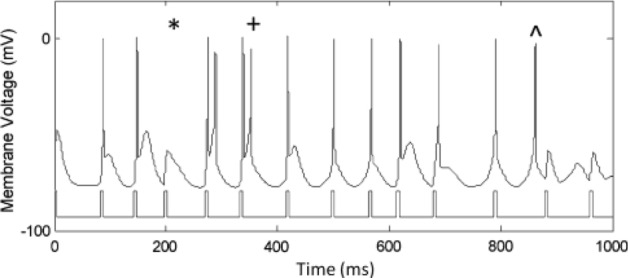
Example of a cell in BG responding to stimulus pulses. Some error responses are highlighted: a miss (*), a burst (+), and a spurious event ($\wedge$)^36^.

In this simulation-based study, we aimed at enhancing the abnormal activation patterns of BG cells due to PD. We considered a mathematical model for BG to analyze the behavior of its cells before and after stimulation as feedback in our algorithm. The mathematical model used in this study has been proposed by So et al.^36^. This method is an updated version of Rubin and Terman mathematical BG model^39^. This BG model is especially used in DBS studies and consists of three basal ganglia nuclei neurons including subthalamic nucleus (STN), globus pallidus pars externa and interna (GPe and GPi) neurons in addition to the thalamic cells (TH). This model considers 100 neurons in each neuronal population. In Fig. 1, an example of different changes in firing pattern is shown in the developed model of So et al.^36^. As shown in Fig. 1, three types of errors consisting of misses, bursts, and spurious events were considered in BG mathematical model developed by So et al.^36^. In the Parkinsonian firing pattern, a miss occurs when a neuron fails to spike, a burst happens when a neuron spikes more than once within 25 ms of a stimulating input, and a spurious event occurs if cell fires in the absence of a stimulating input^36^. All the equations and detailed information regarding the parameters of each cell model used in this study can be found in the Appendix section of the manuscript by So et al.^36^.

In this study, we considered the firing pattern of GPe cells in the BG area. It is known that the membrane voltage of the GPe cells exhibits regular spiking for normal subjects and PD patients who are under the DBS treatment. This cell exhibits bursting behavior when the patient shows physical symptoms^36,37^. By focusing the injected current to STN, the GPe membrane voltage through neuronal connections in the BG network will be affected and its bursting pattern gets restored back to normal regular spiking. The feedback signal in this study includes the spike pattern of GPe neurons extracted from the BG model. Although the recording of this biomarker requires invasive methods, we have utilized its simulated version in this study. In practice, the adaptive algorithm in this study can use other biomarkers such as the hand tremor signal measured non-invasively with the use of an accelerometer sensor^32,40^. In this study, a 130 Hz AC current for each scalp electrode with adjustable amplitudes is chosen similar to DBS method^36,39^.

### Closed-loop system

**Figure 2:**
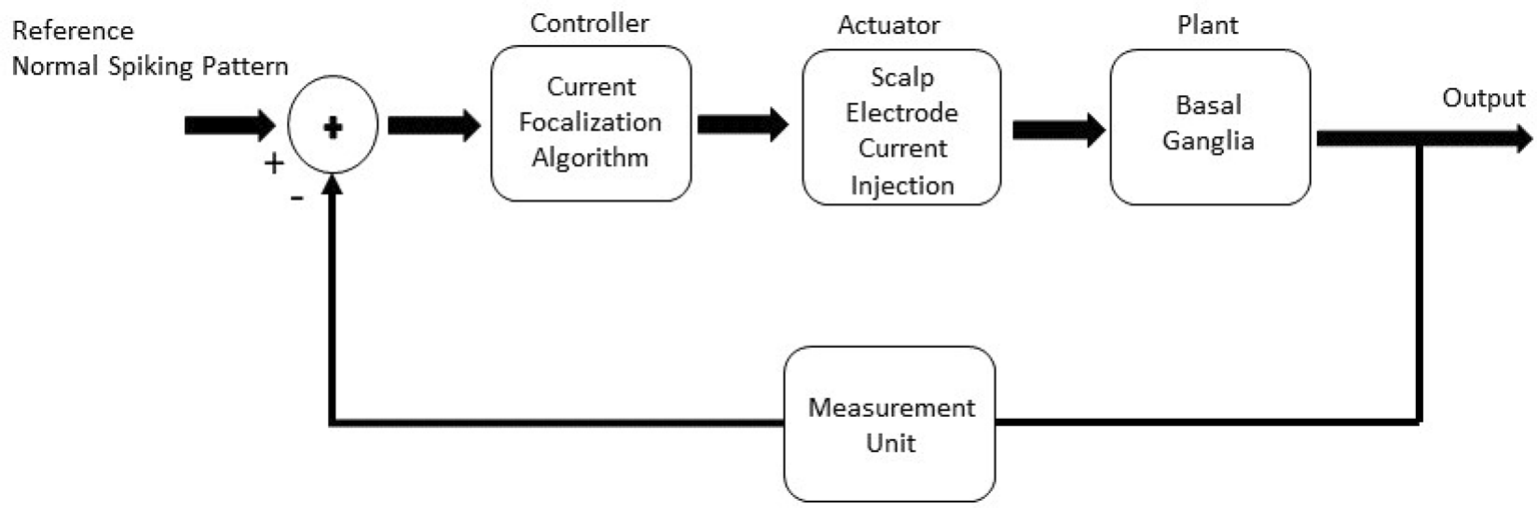
The closed-loop block diagram of the noninvasive transcranial electrical stimulation for PD. By comparing the firing pattern of cells in healthy and Parkinsonian states, an appropriate current injection pattern is calculated to excite the BG and enhance the Parkinsonian firing pattern.

We implemented a closed-loop system to measure changes in the firing pattern of BG cells and enhance the Parkinsonian behavior of the cells in this area. This closed-loop control system is shown in Fig. 2. The system includes a focalization algorithm as a controller, applied currents from the scalp as an actuator, a plant that is the BG mathematical model, and a measurement unit for detecting changes in firing patterns. In our proposed control system the focalization algorithm adjusts the applied current by the scalp electrodes in each iteration. The adjusted applied current delivers the stimulation current to the plant which is the BG mathematical model. The Parkinsonian state is extracted from the BG model. In measurement unit, the Parkinsonian firing pattern is compared with the normal firing pattern. Based on the difference between Parkinsonian and normal state, injected currents (weights of the controller) are adjusted for the next iteration. The gradient vector is determined based on the changes in electrode weights in the previous iteration and the changes in the firing pattern. Using this gradient vector, we can calculate new weights for the electrodes. In real-time experiments, the adaptive algorithm can find the weights of the beamformer to suppress the tremor of hand^30^.

#### Beamforming minimization algorithm

To enhance the Parkinsonian firing pattern by an adaptive closed loop current injection mechanism, we proposed a constraint minimization algorithm based on Least-Mean Squares (LMS) method:

2 $$\begin{aligned} \left\{ \begin{array}{l} {\textrm{Define}}\,\,\,\zeta = \,{\left\| {{\mathrm{d(t)}} - {{\textrm{T}}}{\mathrm{(t,w)}}} \right\| ^2}\\ {\textrm{minimize}}\,\zeta \\ {\textrm{subject}}\,{\textrm{to}}:\,\,\\ \,\,\left| {{w_m}} \right| \le {C_{\max }}\begin{array}{*{20}{c}},&{}{m = 1 \cdots M} \end{array}\\ \sum \nolimits _{m = 1}^M {\left| {{w_m}} \right| } \le {C_{\max }}\, \end{array} \right. \end{aligned}$$

In (2), $\,\zeta$ is the difference between the number of spikes in normal firing pattern ${\mathrm{d(t)}}$ which is changing by time index (t), and the Parkinsonian pattern${{\textrm{T}}}{\mathrm{(t,w)}}$ as a function of the time index (t) and weights of the electrodes (w). ${C_{\max }}$ is a constant representing the maximum allowable current density (identical to 2 mA). In order to make sure that safety limits for injected currents are satisfied, two current constraints are imposed to (2); individual electrode current constraint $\,\,\left| {{w_m}} \right| \le {C_{\max }}$ and total current constraint $\sum \nolimits _{m = 1}^M {\left| {{w_m}} \right| } \le {C_{\max }}$. For simplicity, it is assumed that the current of all electrodes are unity and each current density is applied through a scalar weight value ${w_m},\,\,m = 1,2,\ldots ,M$ as,

3 $$\begin{aligned} {} \textbf{w} = {[{w_1},w_2, \ldots ,w_M]^T}\end{aligned}$$

Where *m* is the number of electrodes. The considered constraints are similar to those used in Dmochowski et al.^24^. Due to inequality constraints this minimization problem (2) has no closed-form solution. Hence, a numerical optimization procedure should be considered. For this purpose, the popular steepest descent method is invoked in (4) while the inequality constraints are enforced at each sample update.

4 $$\begin{aligned} \textbf{w}{\mathrm{(n + 1)}} = {\textbf{w}}{\mathrm{(n)}} - \mu {\nabla _w}\zeta \end{aligned}$$

Where $\mu$ is the step-size parameter which is explained in details in appendix and ${\nabla _w}\zeta$ is the gradient vector given by,

5 $$\begin{aligned} {\nabla _w}\zeta = {\left[ {\frac{{\partial \zeta }}{{\partial {w_1}}},\frac{{\partial \zeta }}{{\partial {w_2}}}, \ldots ,\frac{{\partial \zeta }}{{\partial {w_M}}}} \right] ^T}\end{aligned}$$

The gradient vector is obtained using different methods in simulation or real-time environments. For better understanding, we named these methods as analytical method and perturbation method for using in simulation environment and real-time environment respectively. The analytical method establishes a direct correlation between stimulation current intensity and alterations in the firing pattern, suitable primarily for computational approaches. Conversely, the perturbation method is more attuned to real-time settings. In this approach, the system monitors changes in the designated feedback during each iteration which is alterations in firing patterns in this study, and adjusts the stimulation current accordingly. Both of these methods are mentioned in detail.

***Analytical method***

By applying the chain rule and utilizing the theoretical connections between induced current and the firing pattern, the gradient vector is computed within the simulation-based environment as follows,

6 $$\begin{aligned} \nabla _w\zeta = \left( {\frac{{d{T_r}}}{{d{{\textbf{s}}_{{\textrm{STN}}}}}}} \right) \left( {{\nabla _w}{{\textbf{s}}_{{\textrm{STN}}}}} \right) \end{aligned}$$

where ${{\textbf{s}}_{{\textrm{STN}}}}$ denotes the delivered current density at STN area. Now, the *m*th derivative term of ${{\nabla _w}{{\textbf{s}}_{{\textrm{STN}}}}}$ at the time index *t* is approximated by,

7 $$\begin{aligned} {\left[ {{\nabla _w}{{\textbf{s}}_{{\textrm{STN}}}}} \right] _m} \approx \frac{{\left( {{{\textrm{s}}_{{\textrm{STN}}}}{\mathrm{(t) - }}{{\textrm{s}}_{{\textrm{STN}}}}{\mathrm{(t - 1)}}} \right) }}{{\left( {{{\textrm{w}}_{\textrm{m}}}{\mathrm{(t) - }}{{\textrm{w}}_{\textrm{m}}}{\mathrm{(t- 1)}}} \right) }}\end{aligned}$$

then,

8 $$\begin{aligned} \frac{{d{T_r}}}{{d{s_{{\textrm{STN}}}}}} \approx \frac{{{\mathrm{\Delta }}{{\textrm{T}}_{\textrm{r}}}}}{{{\mathrm{\Delta }}{{\textrm{s}}_{{\textrm{STN}}}}}} = \frac{{\left( {{{\textrm{T}}_{\textrm{r}}}{\mathrm{(t) - }}{{\textrm{T}}_{\textrm{r}}}{\mathrm{(t - 1)}}} \right) }}{{\left( {{{\textrm{s}}_{{\textrm{STN}}}}{\mathrm{(t) - }}{{\textrm{s}}_{{\textrm{STN}}}}{\mathrm{(t - 1)}}} \right) }}\end{aligned}$$

The STN current density (${{\textbf{s}}_{{\textrm{STN}}}}$ ) is considered as the average current densities in FEM nodes associated with the STN area as:

9 $$\begin{aligned} {{\bar{s}}_{{\textrm{STN}}}} = \frac{1}{K}\sum \nolimits _{k = 1}^K {{{\textrm{s}}_{{\textrm{STN}}}}{\mathrm{(k)}}} \end{aligned}$$

**Figure 3:**
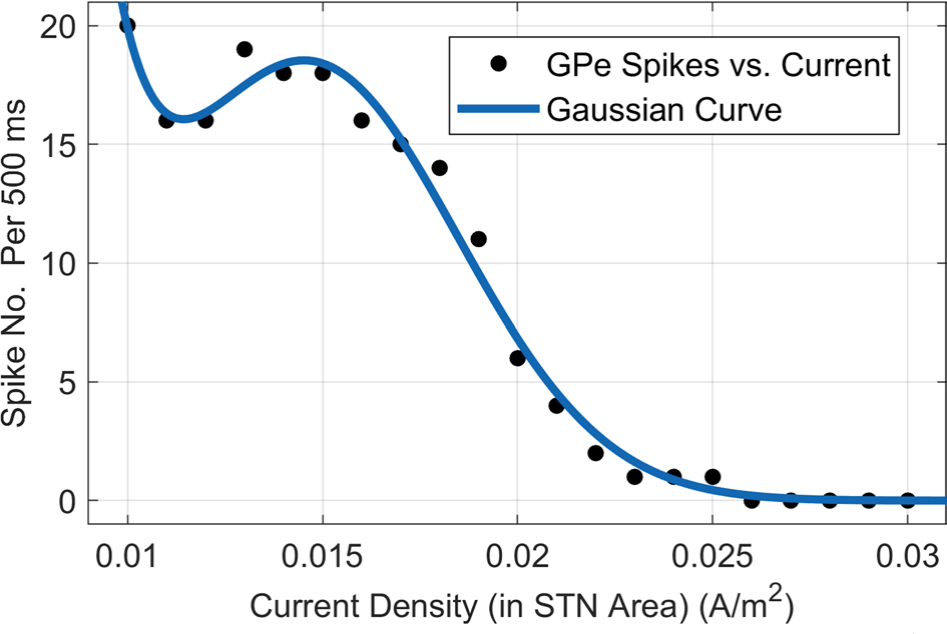
A Binomial Gaussian equation fitted to the GPe spike-Current (density) data obtained from the BG model.

In (9) *K* denotes the total number of FEM nodes of the STN area in the model. This current stimulates the BG and changes the GPe spikes. Therefore, to obtain (6), the relationship between the firing pattern and the STN current (density) based on the mathematical model of the BG should be derived. We fitted a binomial Gaussian curve to the obtained curve depicting the relationship between applied current and changes in the firing pattern of the BG model (Fig. 3). This fitting was achieved by applying an appropriate range of current to the model. The resulting relationship can be expressed as follows:

10 $$\begin{aligned} {T_r} = ae^ {\,\,\left( { - {{\left( {\frac{{{{{\bar{s}}}_{{\textrm{STN}}}} + 0.09}}{0.02 }} \right) }^2}} \right) }+ be^{\,\,\left( { - {{\left( {\frac{{{{{\bar{s}}}_{{\textrm{STN}}}} - 0.015}}{0.005 }} \right) }^2}} \right) }\end{aligned}$$

where *a*, *b*, are $4.8 \times {10^{15}}$ , and 17 respectively. Based on the explored relationship between induced electric field and applied current densities by Dmochowski et al.^24^, we have:

11 $$\begin{aligned} {{\textbf{e}}_{{\textrm{STN}}}}{\mathrm{(k)}} = {{\textbf{A}}_{{\textrm{STN}}}}{\mathrm{(k)}}.{\textbf{w}}\,\end{aligned}$$

where ${{\textbf{A}}_{{\textrm{STN}}}}{\mathrm{(k)}}$ ( $3 \times M$) denotes the stiffness matrix of the STN area whose elements represent the effective resistivity of each FEM node at the spatial coordinate. By putting the current density of STN area (9) in the electric field and the current density relationship ( ${\mathbf{\textit{J}}} = \sigma {\mathbf{\textit{E}}}$), we have:

12 $$\begin{aligned} {{\textbf{s}}_{{\textrm{STN}}}}= {\mathbf{\textit{J}}}= \sigma {\mathbf{\textit{E}}} \end{aligned}$$

By substituting (11) in (12),

13 $$\begin{aligned} {{\textbf{s}}_{{\textrm{STN}}}}= \sigma {{\textbf{A}}_{{\textrm{STN}}}}{\mathrm{(k)}}.{\textbf{w}}\,\end{aligned}$$

We can use (13) for (9),

14 $$\begin{aligned} {{\bar{s}}_{{\textrm{STN}}}} = \frac{1}{{{t_0}}}\sum \nolimits _{t = 1}^{{t_0}} {\sigma \,\left\| {{{\textbf{A}}_{{\textrm{STN}}}}{\mathrm{(t)}}.{\textbf{w}}} \right\| } \end{aligned}$$

Substituting (14) in (10) gives,

15 $$\begin{aligned} {T_r} =&ae^{\left( { - {{\left( {\frac{{\frac{1}{K}\sum \nolimits _{k = 1}^K {\sigma \,\,\left\| {{{\textbf{A}}_{{\textrm{STN}}}}{\mathrm{(k)}}.{\textbf{w}}} \right\| } + 0.09}}{0.02 }} \right) }^2}} \right) }&\nonumber \\&+ be^ {\left( { - {{\left( {\frac{{\frac{1}{K}\sum \nolimits _{k = 1}^K {\sigma \,\,\left\| {{{\textbf{A}}_{{\textrm{STN}}}}{\mathrm{(k)}}.{\textbf{w}}} \right\| } - 0.015}}{0.005 }} \right) }^2}} \right) }&\end{aligned}$$

The gradient vector of (6) could be calculated by finding the gradient of (15). Using the gradient vector in (6), the new weight vector can be found by (4). In the proposed method we found a direct relation (10) between Parkinsonian firing pattern and delivered current density in STN area. We compare the results obtained by this analytical gradient vector calculation with that is found by perturbation method. This comparison will be presented by details in results section. We showed that the results obtained by both methods are the same.

***Perturbation method***

To calculate the gradient vector (5) in a real-time scenario, a perturbation is imposed to each weight ${w_m}$ (perturbation in electrode current). Then the change in the considered biomarker such as tremor of hand is measured which results in $\partial \zeta /\partial {w_m} \approx \Delta \zeta /\Delta {w_m}$. The amount of perturbation must be large enough to create a noticeable change in the biomarker yet small enough not to violate the current constraints. The algorithm tries to find the best distribution of electrode currents to enhance the Parkinsonian firing pattern by directing (focalizing) the currents towards the BG. By applying the perturbations we aim at measuring the sensitivity of the changes in firing pattern to each electrode current. This sensitivity helps us to distribute the currents appropriately between electrodes. Noting that the currents cannot be increased arbitrarily in magnitude due to their constraints. Due to the current constraints the amount of perturbation is dictated by maximum allowable current as given in (2). In fact, one of the three following scenarios may occur:

(I) An individual electrode current exceeds the maximum allowable level but the total electrode current constraint is not violated.
(II) The total current constraint exceeds its maximum allowable level but none of the electrode currents reaches its maximum.
(III) Both the total and single electrode current constraints exceed their limits.

In case I, when the new calculated value for an electrode current exceeds its maximum level, we bring it back to its limit for the next current injection. In case II, all electrode currents are scaled down with identical percentage in order to satisfy the total current constraint. For case III, first we apply the adjustment of case II and then the adjustment of Case I is applied if needed.

Based on two different methods of gradient calculation, weight vector (4) could be found. Using the beamforming minimization algorithm (2), injecting currents to the brain in the closed-loop system changes in each iteration. Optimum weights of the beamformer after enhancing the firing pattern using both methods have been compared and presented in Fig. (7). The purpose of this comparison is to demonstrate the system’s capability to converge, whether in a simulated environment or in a real-life scenario.

**Table 1:** Pseudo-algorithm of the proposed method.

Step 1. Initialization	
Calculate ${{\mathbf{\mu }}_c}$ , (Additional Information 1)	
Set m=1	
Loop1-Start:	
− Apply 2 mA current to the m-th electrode (other electrodes zero)	
− Measure number of GPe spikes and save the result in the m-th element	
− m=m+1;	
Loop1-End	
Normalize ${{\mu }}_{c}$	
Choose Parameters	
Set to a fixed number between 0.05 - 0.001	
Choose the initial weights w(0) (equal amplitudes with the same sign)	
Set the current constraints	
$$\begin{array}{ll} \left| {{w_m}} \right| \le {C_{\max }}\\ \sum \nolimits _{m = 1}^M {\left| {{w_m}} \right| } \le {C_{\max }}\, \end{array}$$	
Step 2. Closed-loop Stimulation	
Apply initial weights, (Section 3.1 )	
Do For Ever:	
− Measure spiking pattern of GPe cells in simulation or hand tremor in real-time experiment	
− Apply the current perturbations and calculate the gradient vector (Analytical method or perturbation method)	
$${\nabla _w}\zeta = {\left[ {\frac{{\partial \zeta }}{{\partial {w_1}}},\frac{{\partial \zeta }}{{\partial {w_2}}}, \ldots ,\frac{{\partial \zeta }}{{\partial {w_M}}}} \right] ^T}$$	
− Find new weights (See Appendix (section Supplementary Information))	
$$\textbf{w}{\mathrm{(n + 1)}} = {\textbf{w}}{\mathrm{(n)}} - \mu \left( {{{\mathbf{\mu }}_c}\,\, \circ {\nabla _w}\zeta } \right)$$	
− Apply the Current Constraints	
−Inject the new Current	

A summary of the closed-loop algorithm and details of steps in each iteration is presented in Table 1.

## Results

In this study two head models are considered; a simple spherical model to determine the parameters of the algorithm, and a realistic MRI-based model to evaluate the performance of the proposed method. Parameters of both head models are given in Table 2. We have provided a detailed presentation of the results of the comparisons and simulations for both models in this section. Specifically, we have discussed various aspects such as the number of electrodes, different initial weight vectors, and optimal current distribution of electrodes based on the spherical head model. Moreover, we have examined the amount of optimum weights using different gradient vectors, the induced electric field in the target area, firing pattern behavior after different iterations, and convergence rate using a realistic head model.

### Spherical head model

The target area in the developed spherical head model is selected as a small sphere in the brain layer with a radius of 1.9 cm roughly equal to the size of a real BG. The location of the target sphere was chosen close to the central line of the two hemispheres to mimic the actual location of the BG^41^. A cube with the length of 0.07 mm was assumed in the target area as a part of STN to calculate its stimulating current^36^. This cube represents just a small part of STN area corresponding to the part considered in BG mathematical model^36^. Due to the small size of the cube the current density and the distributed electric field of the FEM elements in this area are roughly the same. Therefore, the average current density of the STN equals the current density of each FEM node in this area. To avoid lengthy processing time, we used the simple spherical head model for finding the initial weight values and also the best electrode numbers. Therefore, result of both investigations is reported in this section.

**Figure 4:**
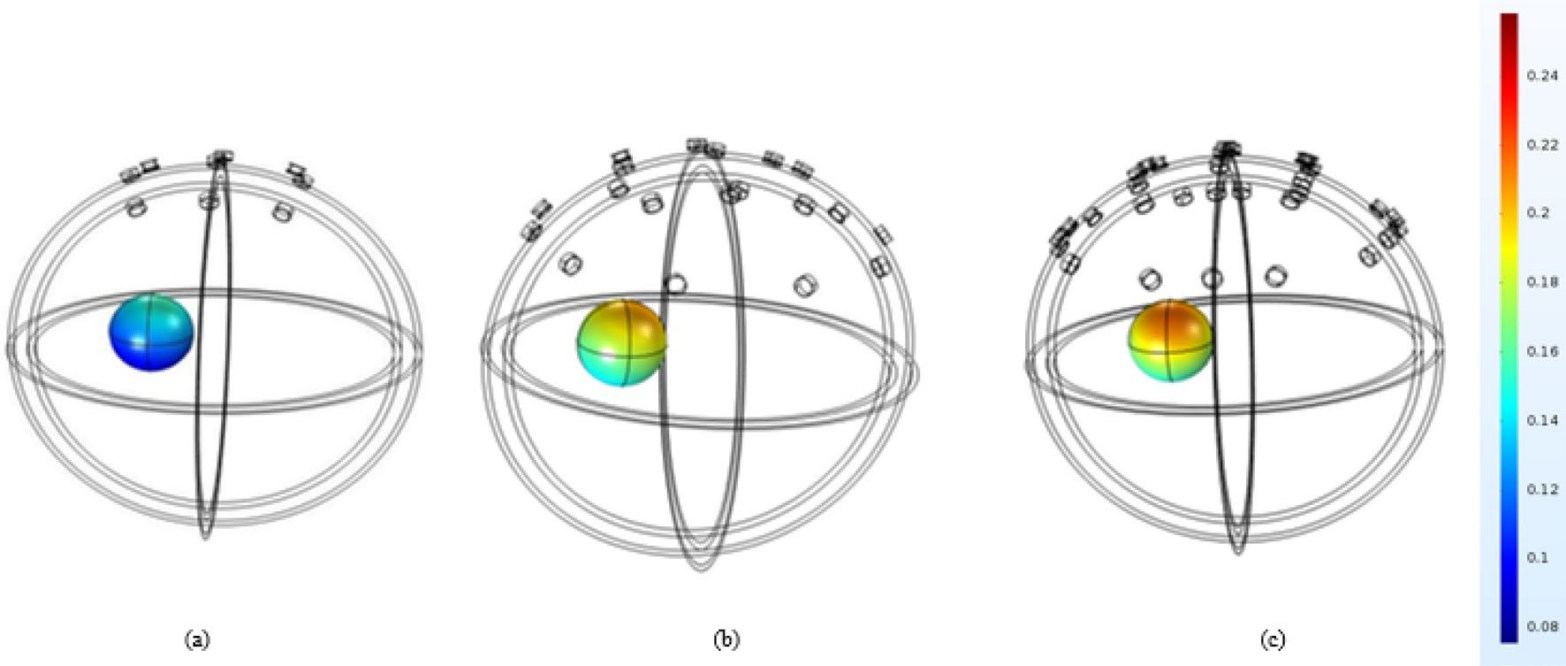
Electric field distribution in simple spherical head model in the target area after current focalization with different number of electrodes, (**a**) 9 electrodes, (**b**) 19 electrodes and (**c**) 32 electrodes. The scale for the numbers are in V/m.

**Figure 5:**
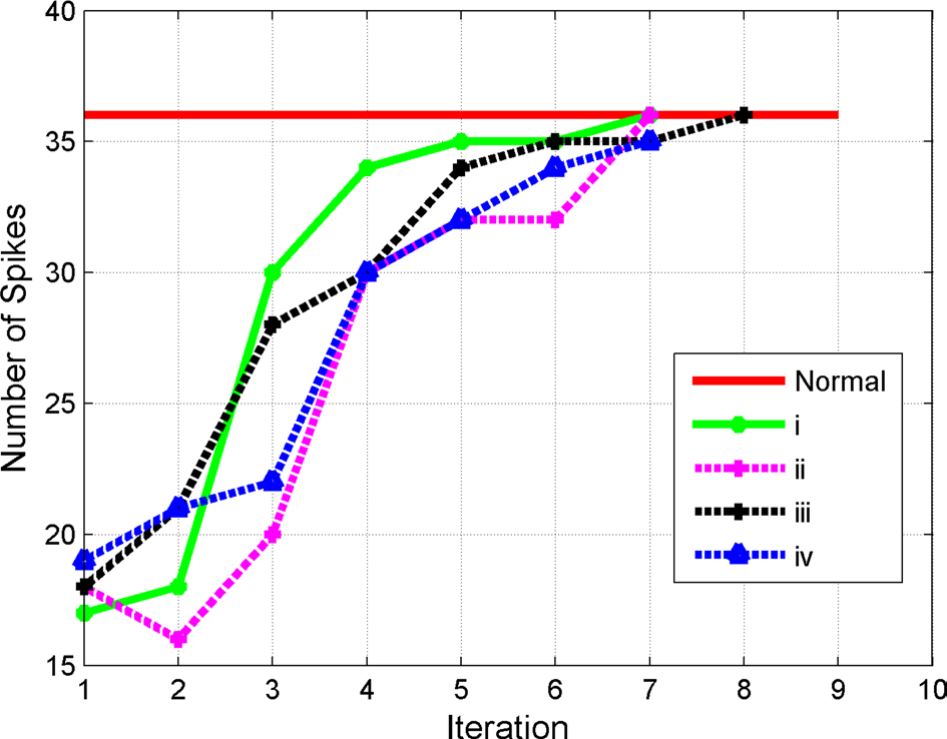
Convergence behavior of four different initial weight vectors (i to iV) as discussed in the text.

#### Number of electrodes

Three different numbers of electrodes 9, 19, and 32 were examined according to the 10–20 standard EEG configuration^42^. For this study, the electrode numbers were selected to encompass a range from the minimum to the possible average number of electrodes utilized in the literature^23,24,34^. As it is shown in Fig. 4, after applying the proposed method, the electric field distributed in the target area with 32 electrodes is higher than 19 and 9 electrodes. However, there is not much difference between the electric field distribution of 19 and 32 electrodes (0.238 and 0.243 V/m respectively). While using more electrodes provide more degrees of freedom for the focalization and slightly better results, it increases computational cost and requires more complex hardware. Thus, we adhere to using 19 electrodes for further simulations.

#### Initial weight vector

In this section we investigated whether the system would converge to the optimal point with different initial weights assigned to the electrodes, using the amplitude of stimulation current as the initial weight vector. Although there is freedom in choosing any initial weight vector within the current constraints, four different initial sets are examined to compare their convergence rates. Suitable initial weight vector leads to faster convergence rate. Considered initial weights include:

1. Equal weights with equal signs (all positive or all negative)
2. Equal weights with arbitrary signs (arbitrary positive or negative numbers)
3. Arbitrary weights with equal signs
4. Arbitrary weights with arbitrary signs

According to Fig. 5, the best initial point is when equal positive currents are assumed for all electrodes as case (1) converges slightly faster than other methods. Despite the nonlinear feature of the problem, using the proposed adaptive procedure ensures convergence of all initial weight vectors. The study considered the healthy state as having a higher number of spikes compared to the Parkinsonian state^36^. If the initial state exhibits a high number of spikes similar to the normal state, the system will remain unaltered. While all cases reach the normal level in the final step, choosing the best initial weight saves processing time.

#### Optimal current distribution of electrodes

**Figure 6:**
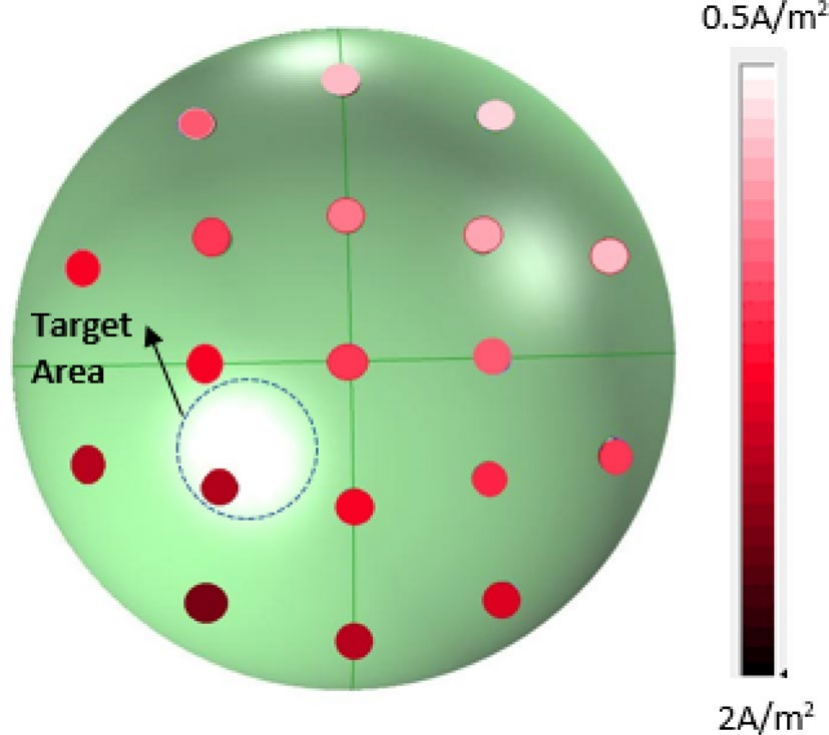
Current density distribution in the simple spherical head model after convergence. Small circles show the location of electrodes on the head. Higher current density is shown by darker colors.

To show the effect of electrode locations with respect to the target point, we illustrated the distribution of optimal weights in Fig. 6. As shown in this figure, electrodes on top of the target point and close to the target area have more contribution than those electrodes further away. This means that the distance of the electrode to the target point is an important parameter for current focalization. The proposed approach seeks to determine the ideal placement of electrodes by adjusting the level of current density applied to each one. This algorithm optimizes both the position of the electrodes and the amount of current density utilized. To identify the most effective electrode placement and current density level, the current delivered to the target region is implemented into a mathematical model of BG during each iteration. Changes in the number of spikes are taken into account as feedback for adjusting and identifying new weights, which produce fresh current densities for each electrode.

#### Optimal weights using analytical and perturbation methods

**Figure 7:**
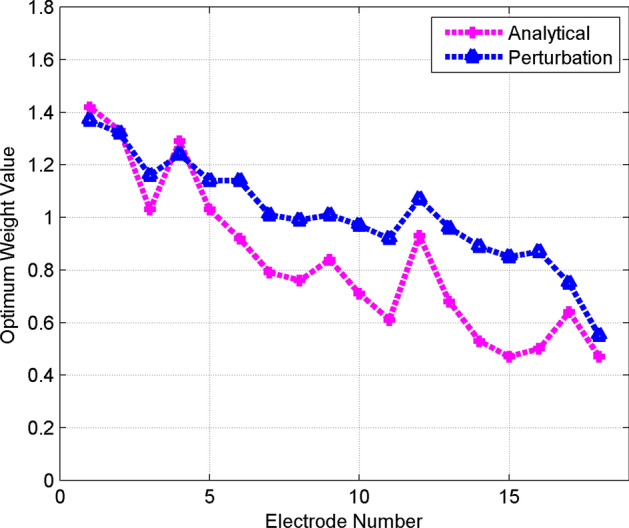
Comparing optimum weight values of electrodes based on analytical and perturbation methods of gradient calculation based on realistic head model. The perturbation method exhibits the same trend as the analytical method.

In the beamforming minimization algorithm (section “Beamforming minimization algorithm”), we explored two different methods for the calculation of gradient vector (5) to use for weight update (4). We introduced an analytical method for gradient calculation based on number of spikes in the firing pattern of the GPe cell and delivered current relation in simulation environment. Moreover, a perturbation-based method is employed for gradient calculation which could be used in an actual experiment. According to Fig. 7, optimum weights obtained by perturbation method is close to the weights found by the theoretical gradient vector. Results show that values of the optimum weight vector are obtained after the same number of iterations. It can be seen that the perturbation-based vector follows closely the theoretical-based gradient vector. The results showed that perturbation method, which is commonly utilized in real-life experiments, functions effectively in the proposed method. In addition this method closely adheres to the optimal weights derived through analytical method. This highlights the adaptable nature of the proposed algorithm, which can be employed in both practical and simulated situations. It is worthwhile mentioning that some error in the magnitude of gradient components can be tolerated as long as the correct direction is preserved. This error in magnitude can be compensated with more number of iterations in the minimization algorithm weight update (4).

**Figure 8:**
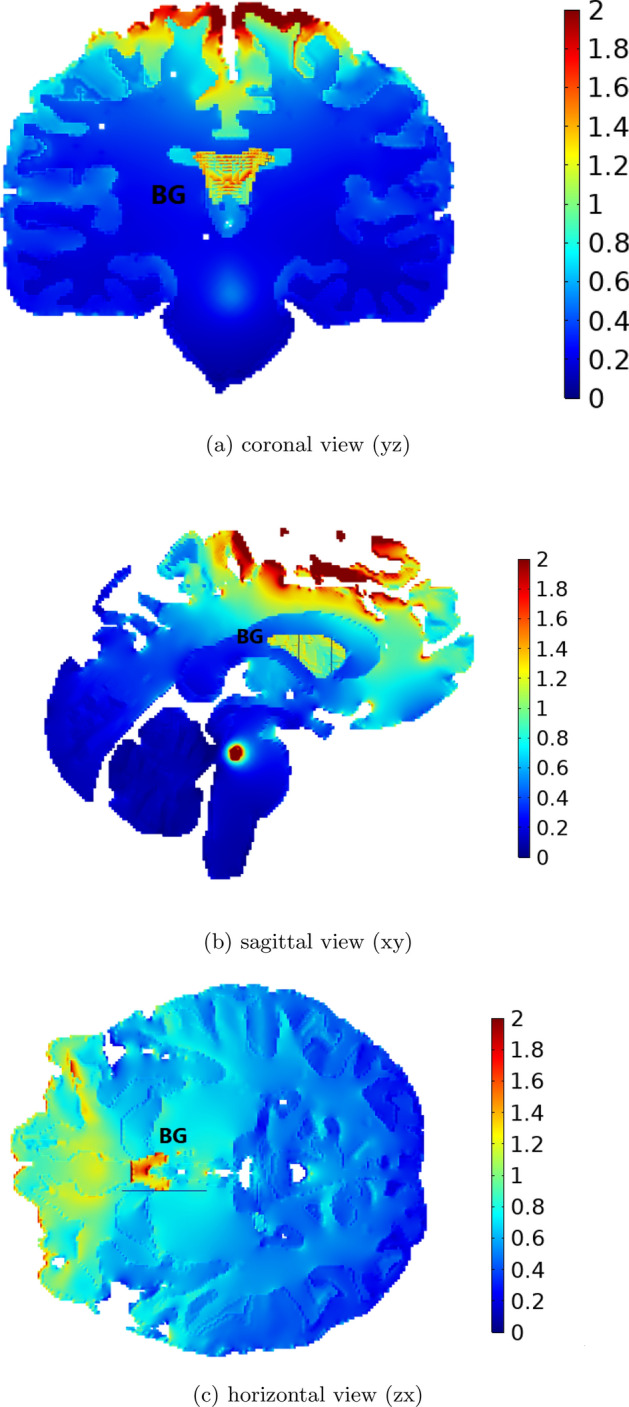
Electric field distribution in the realistic head model with 19 electrodes. Induced electric field in (**a**) coronal view (yz), (**b**) sagittal view (xy), (**c**) horizontal view (zx). Values are in V/m.

**Table 2:** Conductivity and the radius sizes of layers in spherical and realistic head models^24^.

Tissue	Conductivity (s/m)	Radius(cm)
Skin	0.465	9.20
Scalp	0.01	8.95
CSF	1.65	8.30
Brain Tissue	0.2	7.98
Electrodes	5.8$\times$108	0.4
Gray matter (Realistic Head Model)	0.32	NA
White matter (Realistic Head Model)	0.15	NA

NA stands for Not Applicable

### Realistic head model

Conductivity values of different layers for the realistic head model are given in Table 2. The BG area is chosen in its actual anatomical location. The reference electrode is located in F7 based on the 10-20 EEG system. The reference electrode has been utilized as an additional electrode in various setups, acting as the anode in cathodal configurations and as the cathode in anodal configurations. In this study, the realistic head model has been used for validation of the algorithm by presenting the results with a more accurate model of the head. In this section, first, the electric field distribution in head and target area is presented in Fig. 8. Second, we compared our results with previous studies in the field in Table 3. Third, changes in spiking pattern in GPe cell membrane voltage are illustrated before and after stimulation 9. Forth, the behaviour of the proposed algorithm in enhancing the Parkinsonian firing pattern is shown in Fig. 10. Each of these findings has been explained in details.

#### Electric field distribution in target area

Distribution of the electric field is shown in coronal, sagittal, and horizontal views in parts 8a, 8b, and 8c of Fig. 8, respectively. The intensity of the delivered current in the target area is considered for the calculation of the induced electric field in each part^11^. As could be seen in this figure, the induced electric field is considerably focalized in the target area (BG). Moreover, distribution of electric field in areas other than BG is almost close to zero. Consequently, the risk of side effects caused by stimulation of undesirable areas decreases in the proposed algorithm. Since some of the focalization algorithms used in the literature increase the delivered current in the target area without considering the neighboring areas. The intense electric field beneath the surface electrodes is normal and not avoidable due to the nature of the stimulation.

#### Comparison with other studies

To further validate the proposed algorithm we have compared our results with previous studies^21,24,43^ in Table 3. All the selected studies for this comparison have considered non-invasive methods using scalp electrodes. Since there is no difference between the amount of induced electric field using tACS and tDCS in existing computational models, we compared our results with these studies^44^. In the literature, studies focused on the current focalization of non-invasive stimulation methods use different head models and target resolutions. It should be taken into consideration that the results may be impacted by various parameters, including the resolution of the target, the distance between the target and electrodes, and the parameters of the head model. However, to have a better understanding of the performance of the proposed focalization method, studies with head model and target resolution parameters close to those in this study have been chosen. As could be seen, the intensity of the electric field for both the surface and deep target is higher in our method compared to other studies. In alignment with the Vöröslakos et al.^43^, we adjusted the algorithm’s constraints to target 6mA. Our findings revealed a greater induced electric field using the proposed method (3.8 V/m) in contrast to their reported result of 2.9 V/m. Different numbers of electrodes reported in this table show the difference of conventional TES methods with high-definition ones.

**Table 3:** Comparison of our method with previous studies.

Methods	Injected current (mA)	Target area	Maximum induced electric field (v/m)	Number of electrodes
Dmochowski^24^	2	Surface	0.5	64
Huang^21^	2	Deep	1.7	2
Vorloslakos^43^	6	Deep	2.9	12
Proposed method	2	Surface	3.7	19
Proposed method	2	Deep	1.9	19
Proposed method	6	Deep	3.8	19

#### Firing pattern behavior

**Figure 9:**
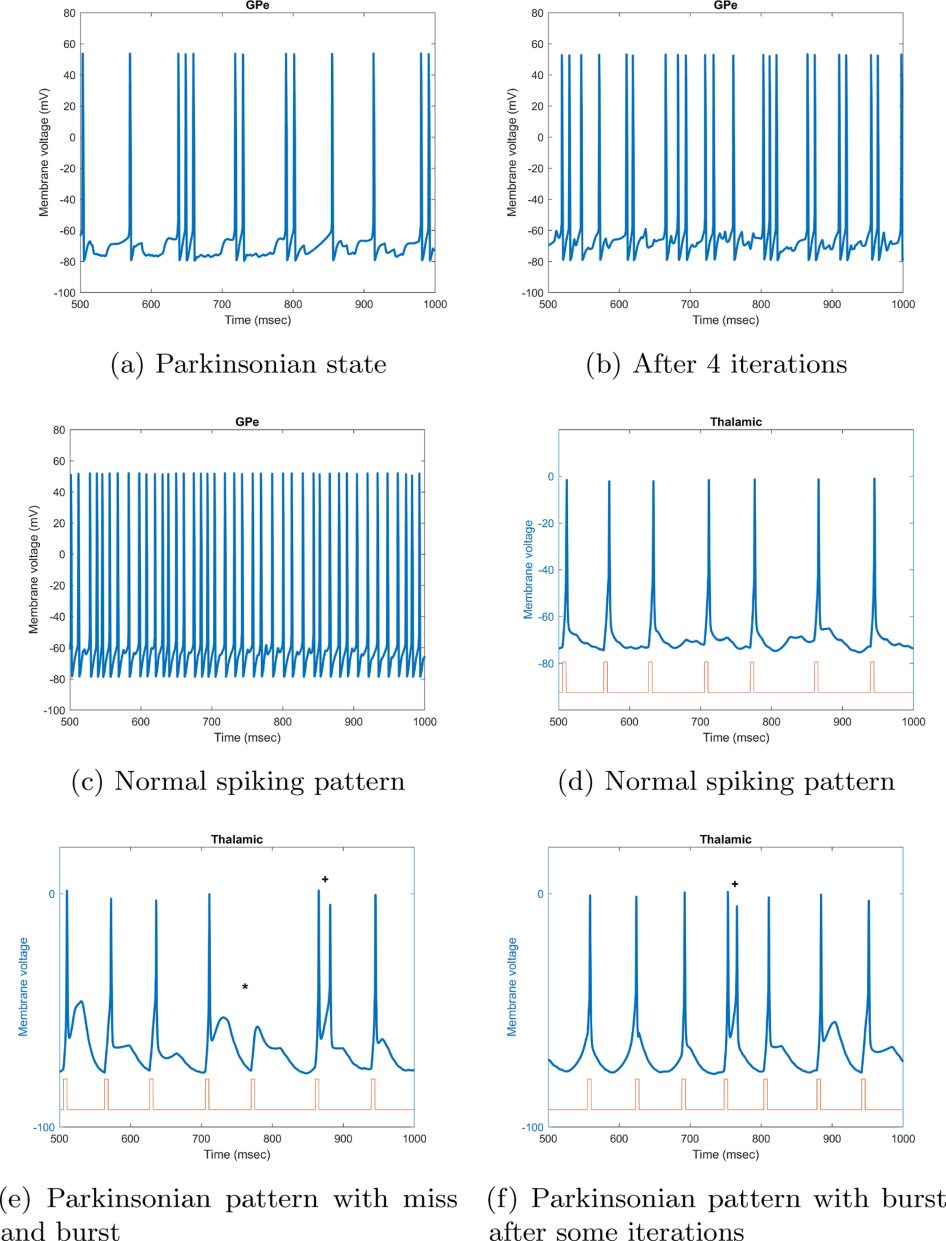
The spiking rate of a typical GPe membrane voltage, (**a**) in Parkinsonian state, (**b**) After 4 iterations of optimization algorithm for focalization of current, (**c**) After convergence of the system and focalizing the current. The spiking pattern of a thalamic cell responding to stimulus pulses from sensorimotor cortex, (**d**) Normal spiking pattern, (**e**) Parkinsonian pattern with miss and burst, (**f**) Parkinsonian pattern with burst after some iterations.

In this study, we considered the changes in the firing rate and pattern of the GPe cells as feedback signal. Different number of spikes in a 500 ms interval is considered as Parkinsonian and healthy states. The evolution of the GPe spiking pattern is shown in Fig. 9. Under the Parkinsonian condition, the firing rates of GPe neurons decreased. While after the completed iteration and stimulation with a focused current, the firing rates of this neuron increased. In a healthy state, more than 36 spikes are observed in this interval which is taken as the desired signal (Fig. 9c). This value drops to around 25 spikes in Parkinsonian state (Fig. 9a). While at the initial point one observes clear Parkinsonian spike behavior, the pattern gradually changes to reach normal in more iterations (Fig. 9b). Since the number of spikes changes in each iteration, we considered the average number of spikes after 10 runs as normal and Parkinsonian states. Regarding the firing pattern, in the healthy condition, the thalamic cells exhibited regular firing at focused stimulation current (Fig. 9d), while in Parkinsonian state, thalamic neurons fired more frequently in a burst-like manner and with misses (Fig. 9e and f).

**Figure 10:**
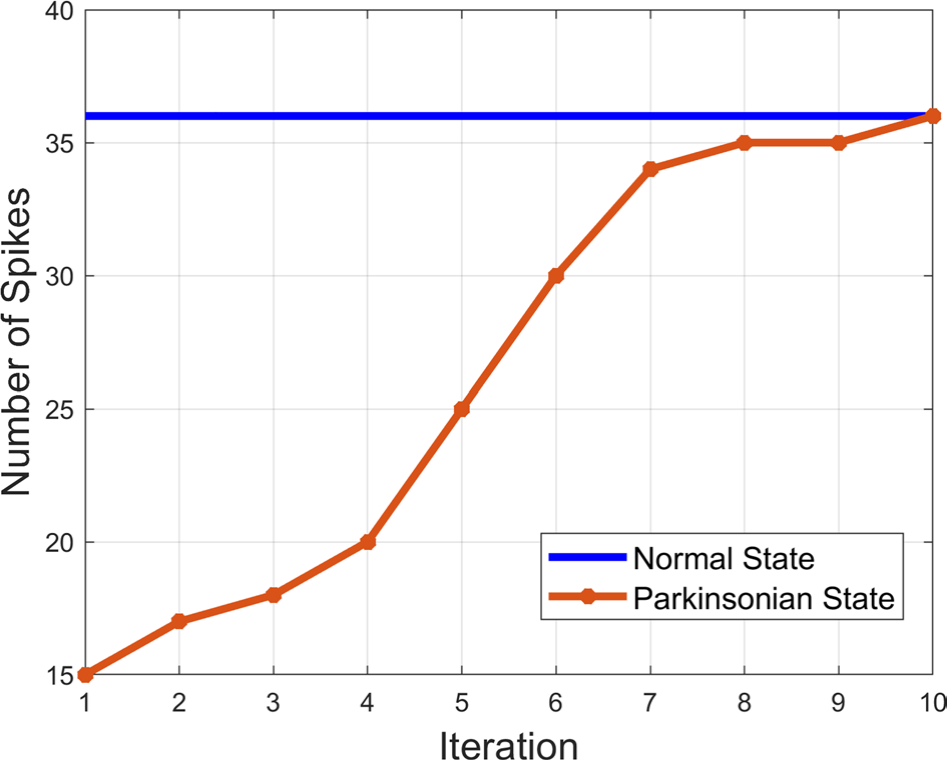
Convergence rate of the system based on changes in the number of spikes in GPe over the course of optimization (realistic head model).

#### Convergence rate

The convergence rate of the system from Parkinsonian to the healthy state is depicted in Fig. 10. Each iteration corresponds to one set of current injection, measurement of changes, and weight vector update through the minimization algorithm (4). As could be seen in this figure, the number of iterations is limited to 10 for convergence. This is a considerable number of iterations for simulation environment. However, more iterations could be expected in an actual experiment due to the factors such as noise, the time-varying behavior of the system, inaccuracy in measurements, and motion artifacts.

## Discussion

This simulation-based study used a closed-loop non-invasive stimulation technique to mitigate Parkinsonian firing pattern in PD. In this study two head models have been utilized; a simple spherical head model, and a realistic MRI-based head model. Using the proposed method, the optimum electrode number and placement, and the maximum induced electric field have been presented for these head models. In the proposed closed-loop system the behaviour of the brain cells in response to stimulation has been considered as the feedback. The spiking pattern of GPe membrane voltage extracted from a mathematical BG model was considered as feedback control^36^. Based on our results, Parkinsonian firing pattern changed to a healthy firing pattern using tACS which is in line with studies in the literature such as^32,45^. In Brittain et al., a closed-loop non-invasive tACS has led to tremor amplitude reduction in PD patients. In a recent study by Schreglmann et al., authors showed that using phase-locked non-invasive stimulation to the tremor oscillation, they suppressed the essential tremor. Although this is a simulation-based study, the real-world experimental applications have been explained in each section. The results showed that using this closed-loop non-invasive method, current was focalized in BG area, and consequently it enhanced the firing pattern of cells in this area.

Considering an adaptive beamforming technique, a minimization algorithm was explored to reduce the difference between Parkinsonian and normal firing patterns in a closed-loop system. We considered the beamforming technique which is used in telecommunications to shape the injecting current in the desired orientation. By finding the best electrode placement on the scalp, the optimum path for current injection is found by the system in each iteration. In addition, due to the adaptive characteristics of the algorithm, this beamforming scheme has the capability to focus the current on two or even multiple targets. Generally, the adaptive feature of the system increases its flexibility in different aspects such as various targets, and number of electrodes. This leads to multiple applications of the system in different diseases and neurological disorders.

The proposed closed-loop system uses a minimization algorithm to find the weights of electrode currents on the scalp. Two safety constraints are considered for each injecting electrode current and total electrode current. For finding the optimum weights of electrode currents, a gradient vector is used to estimate changes in the number of spikes caused by applied currents. Due to inequality safety current constraints (2), there was no closed-form solution for solving the minimization algorithm. We introduced a numerical-based method for gradient vector calculation named as perturbation method^46^. This perturbation-based method could be used in real-time experiments as well. In addition to this method, we explored an analytical method based on the relationship of the applied current and changes in firing pattern for gradient calculation in the simulation environment. We compared the optimum weight values obtained by both of these methods to validate the algorithm. Beamformer weights found by both of these methods were completely close in magnitude in each iteration.

In order to find the optimum results in this simulation-based study, two different head models have been developed, one simple spherical, and one MRI-based realistic head model. Due to the complicated non-linear nature of the brain, no head model can duplicate brain behaviour precisely^29^. However, in brain stimulation studies, the efficacy of head models has been proved already^23,24,33,34^. For instance, in a study by Dmochowski et al.^26^, using MRI-based head models of patients, the optimum electrode placement was found for each participant. Results in this study demonstrated the optimum electrode montage leads to 63% higher induced electric field compared to the conventional approach. In this study, head models in the proposed study were used to (1) validate the adaptive feature of the algorithm in different environments, (2) compare the results obtained by both models, and (3) save the processing time during different findings. In addition, FEM element numbers in these models were chosen to follow previous studies and satisfy the required accuracy for the simulations. As part of the non-invasive closed-loop system, the optimum electrode placement for tACS was determined. The results showed that the proposed algorithm increases the focus of the current in the desired target area by finding the best electrode placement. As indicated in Table 3, our proposed algorithm outperformed other focalization methods used in^21,24,43^. We compared the amount of induced electric field in target areas in both superficial and deep layers of the brain. The induced electric field using the proposed method was higher in both areas compared to other studies. The literature shows that the maximum induced electric field through TES in deep brain layers, such as STN or GPi, is recorded between 0.08 and 5.06 mV/mm through in vivo recording^47,48^. Our simulation-based method results fall within this range, but in actual recordings, this range may vary due to factors like individual patient characteristics. Even though TES generates weaker induced electric fields in deep brain layers than invasive methods, it has been demonstrated that this level can still influence the firing rate of a considerable number of neurons^49,50^. These results validate the performance of the proposed algorithm in finding the best electrode montage for non-invasive brain stimulation.

We have undertaken this study as an initial step to explore the potential application of a closed-loop non-invasive stimulation system for Parkinson’s disease. Despite its purely computational nature and reliance on simulations, our primary aim was to examine whether alterations in electrode count and placement could yield a more precise stimulation within the deeper regions of the brain. When compared to findings in other studies as outlined in Table 3, our study’s results demonstrate that adjusting the stimulation parameters in response to changes in Parkinson’s characteristics could result in more focused stimulation at deeper brain layers. Although it’s important to note that these outcomes may vary in real-time scenarios, they can support the algorithm’s efficacy in a computational environment.

## Limitations of the study

The proposed method and the findings should be interpreted in light of the study’s limitations. First, we linked the explored head models to BG mathematical model in order to extract changes in firing pattern of BG cells caused by PD. Consequently, some inaccuracies may exist in consideration of size and resolution of the target area in the brain and in BG model. However, the size of each considered area was estimated closely to its physiological size and location. Second, the mathematical BG model used in this study has been explored for DBS stimulation. while the AC current with similar characteristics to DBS signal was utilized in our simulations, the neuromodulation effect of tACS on GPe cells was not considered. However, the tACS neuromodulation has been found to have positive effects on PD symptoms^32^. In addition, the adaptivity of the system would preserve its convergence, even in the existence of phase change of the signal. Third, the spiking pattern of GPe membrane voltage was considered as feedback in this study. Although this is a widely-used feedback in DBS studies to separate the Parkinsonian and non-Parkinsonian states, the non-linearity of the brain may cause delays in convergence of the system when tACS is used^36^. In addition, obtaining this biomarker typically demands invasive methods. Although in this study, its simulated version has been employed, in real-world applications, alternative biomarkers, such as the non-invasively measured hand tremor signal with an accelerometer sensor, can be utilized by the adaptive algorithm. Fourth, while this research primarily delved into investigating the computational aspects of closed-loop tACS for Parkinson’s disease, its practical application in real-world scenarios may face constraints. For instance, its prospective implementation might necessitate the consistent use of an easily worn EEG cap, implying a requirement for daily wear. However, the study by Kasten et al.^51^, revealed that even short sessions of 20 min of stimulation effects last up to 70 min, indicating a potential to lessen the need for continuous cap-wearing.

This simulation-based study aimed at investigating the feasibility of having a non-invasive stimulating method to replace the current invasive DBS method for enhancing the Parkinsonian spiking pattern of BG cells in PD. In future studies, the method would be implemented in real-time experiments to test the efficacy of its application in different manners such as tremor suppression in PD.

## Conclusion

A novel non-invasive stimulation method was proposed in this study to affect Parkinsonian spiking pattern of BG cells in PD. By invoking rigorous mathematical modeling and employing various software packages we were able to enhance the Parkinsonian firing pattern of GPe cells by injecting appropriate currents through a high-definition tACS. By using a constraint minimization algorithm, electrode currents are adjusted in a closed-loop mechanism in order to produce a focalized current at the BG. The simulation results confirm our theoretical investigation showing that electrode currents focalized on the BG area. This shows that the proposed method is a promising non-invasive solution to the affect the Parkinsonian symptoms. In a future study the method would be used in a real-world experiment.

### Supplementary Information

Supplementary Information.

## Data Availability

The datasets and codes used and/or analysed during the current study are available from the corresponding author on reasonable request.

## Abbreviations

PD: Parkinson’s disease
DBS: Deep brain stimulation
TES: Transcranial electrical stimulation
BG: Basal ganglia
TC: Thalamocortical
tDCS: Transcranial direct current stimulation
tACS: transcranial alternating current stimulation
UPDRS: Unified Parkinson’s Disease Rating
EEG: Electroencephalography
HD-TES: High-definition TES
MRI: Magnetic Resonance Imagery
FEM: Finite element method
LS: Least Squares
STN: Subthalamic nucleus
GPe: Globus pallidus pars externa
GPi: Globus pallidus pars interna
ROI: Regions of interest
RF: Radio frequency
