# Supplementary material for: Insights from a model based study on optimizing non invasive brain electrical stimulation for Parkinson’s disease
Source: Sci Rep. 2024 Jan 30;14:2447. doi: 10.1038/s41598-024-52355-2 (PMC10828384; doi:10.1038/s41598-024-52355-2)
Supplement: Supplementary file 1 — Supplementary Information. [file 41598_2024_52355_MOESM1_ESM.docx]

**Supplementary Materials:**

Choosing the Step Size Parameter

Since in TES all brain layers are modelled as pure conductive materials, any increase/decrease in each electrode current will have similar effect on the current of the target area regardless of the electrode location. However, in a resistive network closer electrodes to the target area have much higher impact than those further away. Since the amount of injected currents are limited by the given constraints, one needs to give more weights to the electrodes which have higher impacts on the target area. To incorporate this spatial effect of electrodes a contribution percentage vector μc is introduced in weight update equation 4 in the minimization algorithm (2) as,

w(n + 1) = w(n) − μ (μc ◦ ∇wζ) (16)

where ‘◦ ’ denotes the Hadamard (point-wise) multiplication. To calculate μc, we need to determine the percentage contribution of each electrode on the target area by measuring its impact on firing pattern changes. For this purpose, all electrodes are turned off except the one which is set to the maximum allowable current. Then the amount of change in the number of spikes of GPe cells is measured. This process is repeated for all electrodes and corresponding values are recorded in a vector. The normalized version of this vector is defined as μc. This vector is calculated once at the beginning of the procedure.
